# Immunology of Acute and Chronic Wound Healing

**DOI:** 10.3390/biom11050700

**Published:** 2021-05-08

**Authors:** Kamila Raziyeva, Yevgeniy Kim, Zharylkasyn Zharkinbekov, Kuat Kassymbek, Shiro Jimi, Arman Saparov

**Affiliations:** 1 Department of Medicine, School of Medicine, Nazarbayev University, Nur-Sultan 010000, Kazakhstan; kamila.raziyeva@nu.edu.kz (K.R.); yevgeniy.kim@nu.edu.kz (Y.K.); zharylkasyn.zharkinbekov@nu.edu.kz (Z.Z.); kuat.kassymbek@nu.edu.kz (K.K.); 2 Central Lab for Pathology and Morphology, Faculty of Medicine, Fukuoka University, Fukuoka 814-0180, Japan; sjimi@fukuoka-u.ac.jp

**Keywords:** acute wound, chronic wound, cutaneous wound healing, innate immunity, adaptive immunity

## Abstract

Skin wounds greatly affect the global healthcare system, creating a substantial burden on the economy and society. Moreover, the situation is exacerbated by low healing rates, which in fact are overestimated in reports. Cutaneous wounds are generally classified into acute and chronic. The immune response plays an important role during acute wound healing. The activation of immune cells and factors initiate the inflammatory process, facilitate wound cleansing and promote subsequent tissue healing. However, dysregulation of the immune system during the wound healing process leads to persistent inflammation and delayed healing, which ultimately result in chronic wounds. The microenvironment of a chronic wound is characterized by high quantities of pro-inflammatory macrophages, overexpression of inflammatory mediators such as TNF-α and IL-1β, increased activity of matrix metalloproteinases and abundance of reactive oxygen species. Moreover, chronic wounds are frequently complicated by bacterial biofilms, which perpetuate the inflammatory phase. Continuous inflammation and microbial biofilms make it very difficult for the chronic wounds to heal. In this review, we discuss the role of innate and adaptive immunity in the pathogenesis of acute and chronic wounds. Furthermore, we review the latest immunomodulatory therapeutic strategies, including modifying macrophage phenotype, regulating miRNA expression and targeting pro- and anti-inflammatory factors to improve wound healing.

## 1. Introduction

Skin wounds have a tremendous negative impact on healthcare systems and the economy worldwide. It was reported recently that nearly one billion people suffer from acute and chronic wounds globally [1]. This enormous number translates into huge financial expenditures [2]. It was estimated that in developed countries, the expenditures associated with chronic wound management accounted for as much as 3% of total healthcare spending [3]. For instance, in the United States, the overall costs associated with chronic wounds is approximated to be 50 billion USD per year [4]. The situation is likely to be aggravated by low healing rates. In fact, it was reported that publicly available healing rates for skin wounds were significantly overestimated. In particular, the data from randomized-controlled studies provides an average healing rate of 40%, whereas the reported rate is usually greater than 90% [5].

The classification of skin wounds into acute and chronic is based on the pathogenesis and consequences [6]. Acute wounds undergo a series of molecular events that eventually result in the regaining of structural integrity. By contrast, chronic wounds fail to resolve and are characterized by pathologic processes, such as continuous inflammation, persistent infections and necrosis. Generally, four overlapping phases are identified in acute wound healing, namely, hemostasis, inflammation, proliferative phase and remodeling [7]. When an acute wound occurs, the very first response is hemostasis, which terminates bleeding and prevents blood loss. During the inflammatory phase, the injury to the skin activates an elaborate immune response that destroys the pathogens entering the wound and prepares the tissue for the restoration of anatomical integrity. The latter occurs in the proliferative phase and involves the formation of granulation tissue, neovascularization and re-epithelialization [4,8]. Finally, acute wound healing concludes with the remodeling phase, during which the granulation tissue is substituted with a scar and the epidermis is freed from immune cells, which either die by apoptosis or relocate to the dermis [6,8].

Immune cells and factors are the key regulators and players in acute wound healing [9]. Neutrophils and basophils are the first responders to injury. In addition, other innate and adaptive immune cells, such as macrophages, mast cells, Langerhans cells (LCs), T cells and B cells, were shown to be involved in the process [10]. Dysregulation of the immune response during wound healing leads to the emergence of chronic wounds [11]. In chronic wounds, the inflammatory phase fails to resolve, leading to poor and delayed healing [6,12]. Persistent inflammation in such wounds is characterized by several features. Specifically, there are excessive quantities of pro-inflammatory macrophages, whereas the amount of macrophages with anti-inflammatory phenotypes is low [13,14]. Furthermore, macrophages found in chronic wounds have limited capability to clear dead neutrophils. This leads to the establishment of a highly inflammatory environment with an overabundance of inflammatory mediators, such as tumor necrosis factor-α (TNF-α) and interleukin-1β (IL-1β) [14]. Moreover, chronic wound macrophages release several matrix metalloproteinases (MMP), namely MMP-2 and MMP-9, which degrade the extracellular matrix (ECM) and prevent the commencement of the proliferative stage of healing.

Management of chronic wounds still remains an issue since continuous inflammation in these wounds is very difficult to control. One reason for this is the formation of bacterial biofilms on the surface and within chronic wounds. Biofilms interact with the host immune system by activating neutrophils and pro-inflammatory macrophages, resulting in the accumulation of inflammatory cytokines such as TNF-α and IL-6, as well as MMP [12]. On the other hand, the dysregulated immune environment of chronic wounds supports the proliferation of bacteria, leading to the vicious cycle of biofilm growth and continuous inflammation. Moreover, biofilms are very difficult to eradicate therapeutically due to reduced penetration of antimicrobial agents into the biofilm, presence of multiple microbial species, rapid development of antibiotic resistance by the biofilm bacteria and many other challenges [15]. Several strategies have been proposed to improve the healing of chronic wounds. They can be generally classified into biologic agents, biomaterials and cell-based strategies [16]. Bioactive molecules that stimulate neovascularization and re-epithelization have shown positive results in pre-clinical studies. In addition, they can be combined with biomaterials, which could improve their half-life and promote controlled release. On the other hand, biomaterials can be used on their own to provide physical protection to the injured skin. Another therapeutic strategy for wound healing is cell-based technologies using bone marrow-derived mesenchymal stem cells, adipose-derived cells, epidermal cells and others. Multiple studies demonstrated that cellular therapies improved wound healing by enhancing angiogenesis and re-epithelization. Importantly, the aforementioned strategies can be used for immunomodulatory purposes in wound healing. As was discussed above, the immune system is a key contributor to the resolution of acute wounds as well as the persistence of chronic wounds. This provides rationale for utilizing immunomodulation to improve the healing of acute and chronic wounds. To date, multiple immunomodulatory strategies have been proposed for skin wound repair [6]. They include cell-based strategies, molecular therapies and biomaterial-based approaches [17,18,19,20,21,22,23]. In this paper, we review the role of an innate and adaptive immune system in the pathogenesis of acute and chronic wounds and discuss the latest advances in the application of immunomodulatory therapies to improve wound healing.

## 2. Acute Wound Healing

Cutaneous wound healing is a complex process involving various immune and structural cells, whose secretion of cytokines, chemokines and growth factors orchestrate the phases of healing. Typical wound healing is traditionally divided into four sequential stages: hemostasis, which lasts from minutes to hours after skin damage, acute inflammation, which takes 1 to 3 days, proliferation, which usually lasts from a few days to a month, and finally, skin remodeling or scar formation [8,10]. Tissue damage is followed by rapid vessel contraction to prevent exsanguination from vascular injury [24]. Platelets, the primary cellular mediators of hemostasis and coagulation, initiate healing immediately after they receive signals from ECM and local resident cells [25,26]. The ECM secretes proteins, such as fibronectin, collagen and von Willebrand factor, that attach to platelet receptors, such as glycoprotein 6, leading to the formation of clots [24]. A blood clot is composed of platelets inserted in a net of fibrin, fibronectin, vitronectin and thrombospondin and is crucial for protection from bleeding [27,28]. Apart from that, a blood clot defends an injured environment from bacterial invasion and serves as a source of cytokines and growth factors that are released from activated platelets and required for immune cell recruitment [27].

The degranulation of α-granules from platelets leads to the release of certain factors, including platelet factor 4 (PF4), platelet-derived growth factor (PDGF), vascular endothelial growth factor (VEGF), fibroblast growth factor (FGF), hepatocyte growth factor and transforming growth factor-β1 (TGF-β1), which are crucial for the initiation of inflammatory processes [29,30,31]. The most abundant component of α-granules, PF4, facilitates neutrophil and macrophage recruitment, monocyte differentiation and reactive oxygen species (ROS) formation [30]. Platelet-derived TGF-β1 stimulates keratinocyte proliferation as well as remodeling and regeneration of the epidermal layer [32]. Finally, PDGF induces chemotaxis and proliferation of immune and structural cells and promotes the formation of new blood vessels and granulation tissue [31]. Recent data shows that the inhibition of PDGF-AA in adipose-derived stem cells upon transplantation into injured murine skin reduced the positive effect on wound healing [33]. In addition, during degranulation, platelets release pro-inflammatory factors, such as IL-8, C-X-C motif chemokine ligand 8 (CXCL8), IL-1α, IL-1β, IL-6 and TNF-α, that promote the mobilization of the complement system [8]. An activated complement system induces macrophage, neutrophil and mast cell infiltration through the secretion of anaphylatoxins, such as C3a and C5a [34]. Anaphylatoxins also facilitate vascular permeability, further enhancing inflammatory cell infiltration [35].

After skin injury, acute inflammatory response is activated by damage-associated molecular patterns (DAMPs), cellular Ca^2+^ waves, release of ROS, lipid mediators and chemokines [4]. Necrotic and apoptotic cells release DAMPs, such as high mobility group box-1 (HMGB1), S100 proteins, heat shock proteins, IL-1α, IL-33 and extracellular cleavage products (i.e., hyaluronan and fibronectin) [36,37,38]. DAMPs can activate various pathogen recognition receptors, including Toll-like receptors (TLRs), on the surface of residential monocytes/macrophages, neutrophils, dendritic cells (DCs), T cells, mast cells and keratinocytes [39].

Keratinocytes play an important role in promoting the initiation of the inflammatory phase. They express different TLRs, such as TLR-3, TLR-4 and TLR-9, which are upregulated in acute wounds [40,41]. TLRs expressed on the surface of keratinocytes recognize DAMPs released from injured cells, which result in the activation of the receptor itself and subsequent expression of cytokines (interferon-β (IFN-β), TNF-α, IL-8, IL-18 and IL-36γ) and chemokines (C-C motif ligand (CCL) 20 and CCL27) [40]. Particularly, TLR-4 activates the TLR4/p38 and JNK MAPK signaling pathways, resulting in the stimulation of inflammatory cytokine production [41]. In addition, an increase of intracellular Ca^2+^ at the edge of a wound occurs within the first several minutes after skin injury and can contribute to inflammation and cytokine release [4]. Moreover, one of the ROS, hydrogen peroxide, is rapidly released by damaged cells in the wound site and can lead to a decrease in the risk of infections, activation of keratinocyte regeneration and recruitment of neutrophils to the site of the wound [4].

**Figure 1:**
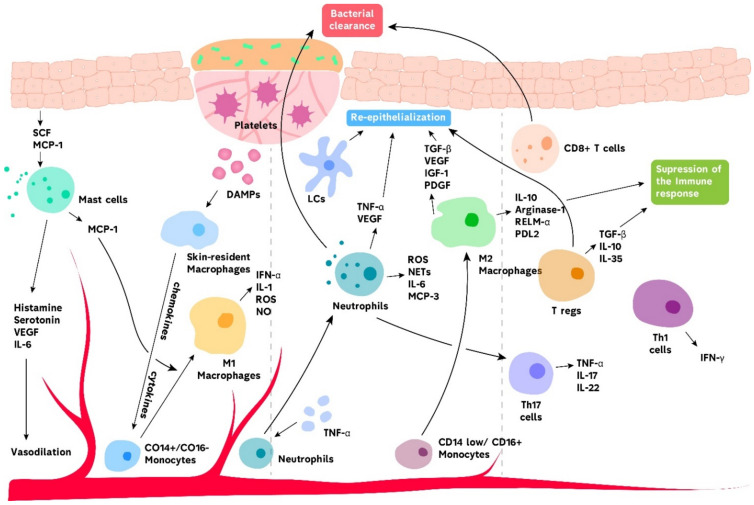
Immune system in acute wound healing. Skin-resident macrophages are the first immune cells that respond to injury. When activated by DAMPs, they release cytokines and chemokines to recruit neutrophils and monocytes to the inflammatory site. Monocytes then differentiate into pro-inflammatory M1 macrophages. Mast cells also facilitate monocyte differentiation by secreting monocyte chemoattractant protein-1 (MCP-1). Moreover, mast cells, which are stimulated by keratinocytes, secrete mediators to promote vasodilation that enhance immune cell recruitment. Activated neutrophils release ROS, neutrophil extracellular traps (NETs), cytotoxic granules and other mediators that promote bacterial clearance and tissue re-epithelialization. Pro-healing M2 macrophages contribute to tissue repair and inhibition of inflammation. LCs promote tissue repair, although the precise mechanism is not clear. Various T cells subsets are found in acute wounds and are responsible for bacterial elimination, modulation of immune responses and tissue remodeling.

The inflammation phase is characterized by the presence of neutrophils and pro-inflammatory macrophages at the site of injury (Figure 1). In this step, the functions of these cells are mainly aimed at cleansing wounds from infections and removing cell debris. The proliferation phase of wound healing involves keratinocytes, fibroblasts, macrophages and endothelial cells, whose active cooperation promotes re-epithelization, angiogenesis and fibroplasia [24,34,42]. This phase is characterized by the presence of pro-healing subsets of immune cells, including M2 anti-inflammatory macrophages and T regulatory cells (Tregs). Tissue remodeling may last up to several years and mainly involves fibroblasts that are responsible for the replacement of fibrin clot with scar tissue, slowing the angiogenesis and changing the collagen composition [24]. Overall, acute wound healing starts with the recruitment of platelets that form blood clots in the damaged area and secrete signaling molecules to attract innate immune cells and factors to promote vascular permeability. Neutrophils and pro-inflammatory macrophages clear the wound from bacterial infection and eliminate debris and dead cells through phagocytosis. This is followed by alteration from pro-inflammatory to pro-healing immune response to inhibit the inflammation and initiate the tissue remodeling process.

### 2.1. Innate Immunity in Acute Wound Healing

#### 2.1.1. Basophils and Mast Cells

Basophils are one of the first cells that infiltrate into the injured tissue attracted by endogenous factors, such as IL-33, IL-18, thymic stromal lymphopoietin (TSLP), granulocyte-macrophage colony-stimulating factor (GM-CSF), IL-3, IL-7, TGF-β, VEGF and IFN type I [43]. They are the major producers of IL-4, the cytokine which is known to facilitate fibroblast proliferation and chemotaxis as well as the production of collagen and ECM molecules in skin wounds [44,45].

Mast cells play a multifunctional role in the inflammatory phase and can be recruited to the site of the wound through release of stem cell factor (SCF) and monocyte chemoattractant protein (MCP-1) by keratinocytes [34]. Their degranulation results in the release of mediators that induce vasodilation, such as histamine, serotonin, TNF, VEGF, IL-6, IL-8 and proteases, into the damaged environment. Mast cells also release MCP-1, which stimulates monocytes differentiation into fully phagocytic cells. In addition, the secretion of IL-4 promotes enhanced proliferation of fibroblasts [34,46,47]. The secretion of VEGF and basic FGF (bFGF) promotes enhanced proliferation of fibroblasts and keratinocytes. At the same time, it appears that mast cells are not a significant source of proangiogenic mediators and further the proliferative response in a healing wound but are significant contributors in the recruitment and accumulation of neutrophils at the wound site [48]. Furthermore, mast cells contribute to enhanced scarring after skin injury and may initiate transition from scarless to fibrotic healing in mature skins [49]. Recent findings revealed the crucial role of zinc in mast cell-mediated wound healing. Zinc and mast cells promote the secretion of IL-6, which is required for normal wound repair from inflammatory cells via the GPR39-mediated PKC/MAPK/C/EBP pathway [50]. Furthermore, IL-6 induces thrombus resolution by enhancing the gene expression of PLAU, MMP2 and MMP9 in macrophages via the signal transducer and activator of transcription 3 (STAT3) signaling pathway [51]. Finally, mast cells serve as antimicrobial and antiviral protectors in the damaged tissue. The treatment of infected murine skin with mastoparan, an activator of connective tissue mast cells, reduced *Staphylococcus aureus* infection and increased tissue repair by enhancing the activation and migration of CD301b^+^ DCs and neutrophils [52].

#### 2.1.2. Neutrophils

Neutrophils, as “first responders” for tissue injury, accumulate at the wound site at an early stage of inflammatory response and remain the most abundant cell type during the first 24 h [7,53]. Neutrophils can be recruited to the site of injury by DAMPs, pro-inflammatory cytokines, including TNF-α and chemo-attractants, such as CXCL 1–3 and IL-8, anaphylatoxins C3a and C5a and macrophage inflammatory protein-1α [54]. The main role of neutrophils in skin injury is to prevent bacterial invasion due to high efficiency in pathogen clearance by releasing ROS and cytotoxic granules and forming neutrophil extracellular traps (NETs) with subsequent phagocytosis of pathogens (Figure 1) [55]. Furthermore, neutrophils amplify inflammation by releasing cytokines and chemokines, such as TNF-α, IL-1β, IL-6, CXCL8, CXCL2 and MCP-1 [53]. These factors attract more neutrophils as well as macrophages and T cells. In addition, they can possess anti-pathogenic functions by expressing antimicrobial agents and proteases and secreting multiple growth factors and cytokines. Moreover, neutrophils induce keratinocyte and fibroblast proliferation [56]. All these actions lead to the clearance of cellular debris and initiate the rapid healing process after first response [4,53]. Particularly, neutrophils produce leukotriene B4 and undergo vital NETosis, which results in phagocytosis of bacteria, dead erythrocytes and cellular debris. Furthermore, neutrophils modulate inflammation by producing ROS, different cytokines (IL-1β, IL-6, IL-10) and MCP-1, which attract more immune cells including monocytes and macrophages [54].

Neutrophils can direct an adaptive immune response by presenting antigen to T cells [57]. In addition, a recent study found that neutrophil antimicrobial peptide cathelicidin can promote T cell differentiation towards Th17, which in turn positively impacts wound healing [58,59]. Migration and activation of neutrophils result in the clearance of DAMPs and the production of lipid mediators and anti-inflammatory cytokines, such as IL10 and IL-1Ra, leading to a decrease in inflammation at the wound site. Neutrophils can also degrade pro-inflammatory cytokines by NETs, release soluble factors and modulate polarization of macrophages towards M2 phenotype. Activated neutrophils might initiate wound closure, re-epithelialization and formation of new vessels in the wound site by expressing cytokines and growth factors, such as TNF-α and VEGF [54]. For successful wound healing and reduction of inflammation, clearance of neutrophils is also required [60]. A study in murine models demonstrated that cutaneous wounds heal faster in neutrophil-depleted mice compared with controls, concluding that excessive recruitment of neutrophils at the wound site may delay wound healing [61]. Macrophages can phagocyte neutrophils through β2 integrins after completion of DAMP clearance [54,62]. Furthermore, matricellular protein CCN1 also plays an important role in neutrophil clearance. It binds directly to TLRs and induces cytokine expression and controls neutrophil infiltration through the MyD88 signaling pathway [63]. Moreover, it seems that CCN1 can act as a bridging molecule between neutrophils and macrophages. CCN1 can bind to a neutrophil cell membrane phospholipid called phosphatidylserine, which is a common “eat me” signal on apoptotic cells, and presents it to integrins on macrophages, activating efferocytosis of neutrophils. This leads to the clearance of neutrophils from the wound site and contributes further to the wound healing process [64]. Taken together, neutrophils play a pivotal role in the response to skin injury, initiating their protective functions by releasing pro-inflammatory and anti-pathogenic factors, and their subsequent production of anti-inflammatory cytokines and clearance by macrophages, thus leading to proper wound healing.

#### 2.1.3. Macrophages

It is well established that skin macrophages play a crucial role in acute wound healing [14,65]. Using several animal skin wound models, it was demonstrated that the depletion of macrophages led to delayed or impaired healing. It resulted in reduced ECM reformation, decreased angiogenesis and delayed wound closure [14]. Skin has two subsets of macrophages, namely, resident macrophages, which develop from the yolk sac and reside in the dermis, as well as bone marrow-derived circulating monocytes [14,66]. Skin-resident macrophages express CD64^+^, MERTK^+^ and CCR2^−/low^ surface markers and are capable of self-renewal. When an acute injury occurs, dermal macrophages are one of the first cells to respond. Specifically, they recognize DAMPs and secrete chemokines that recruit neutrophils and monocyte-derived macrophages from the blood [14]. Notably, the response of resident macrophages to skin injuries is only short-term, and later stages of inflammation are propagated by monocytes recruited from the circulation. Blood monocytes that are attracted to wounds are classified into classical or pro-inflammatory CD14^+^CD16^−^ monocytes and anti-inflammatory CD14^low/−^CD16^+^ monocytes. The former type is more capable of differentiating into M1 macrophages, which propagate inflammation, while the latter subset of monocytes gives rise mostly to reparative M2 macrophages (Figure 1). Nevertheless, as with other tissues, an attempt to describe skin macrophages as M1 and M2 phenotypes is probably an oversimplification since a wider range of phenotypes exist [67,68].

In their recent review dedicated to the role of macrophages in wound healing, Krzyszczyk and colleagues classified wound macrophages into three types based on their functions during healing, namely, pro-inflammatory, pro-wound healing and pro-resolving macrophages [14]. Pro-inflammatory macrophages produce inflammatory mediators, such as nitric oxide, ROS, IL-1, IL-6 and TNF-α, as well as MMP-2 and MMP-9. Pro-wound healing macrophages are responsible for tissue repair and neovascularization, which is mediated by PDGF, insulin-like growth factor 1 (IGF-1), VEGF and TGF-β1. The release of IL-10 and PDGF-β by wound-associated macrophages induces epithelial repair [8,69]. Importantly, this subset of macrophages inhibits the actions of MMPs. Finally, pro-resolving macrophages possess immunoregulatory properties, i.e., they inhibit inflammation and immune cells by secretion of IL-10, arginase 1, resistin-like molecule-α, programmed death ligand 2 and TGF-β1 [14]. Besides the aforementioned phenotypes, very recent research by Xu and colleagues has demonstrated a unique subtype of macrophages which forms when skin macrophages engulf apoptotic endothelial cells [70]. These macrophages possess features of both M1 and M2 phenotypes. Taken together, skin macrophages actively participate in all stages of acute wound healing and are essential for proper wound resolution.

#### 2.1.4. Langerhans Cells and Dendritic Cells

Langerhans Cells (LCs) are unique antigen-presenting cells, which reside exclusively in stratified squamous epithelia. Ontogenically, LCs are related to macrophages, but they also share similarities with conventional DCs [71]. Thus, LCs express Mafb transcription factor, which is specific to a common macrophage progenitor, as well as Zbtb46 transcription factor, which is a marker of DCs [72]. Skin LCs are localized mainly in the epidermis, where they are the only APCs making up 3–5% of nucleated cells [71]. Yolk sac macrophages and fetal liver monocytes give rise to resident LCs, which are capable of self-renewal [72]. During skin injury and inflammation, however, epidermal LCs are largely replenished by circulating monocytes. Murine LCs are characterized by CD11b^+^CD11c^+^F4/80^+^MHCII^+^ phenotype [65]. Other important markers of skin LCs are langerin (CD207) and the epithelial cell adhesion molecule (EpCAM, CD236) [72]. Langerin is a C-type lectin receptor that is responsible for the formation of LC-specific endosomal organelles called Birbeck granules [71,72]. It is important to note that langerin is not unique to LCs and is expressed on dermal DCs as well [73], whereas EpCAM is in turn involved in LCs migration to the lymph nodes [72].

There is a limited amount of compelling data regarding the role and importance of LCs and DCs in acute wound healing in the skin. The two most recent studies provide contradictory answers to this question. Rajesh and colleagues demonstrated that the depletion of langerin+ cells with diphtheria toxin led to a smaller wound size as well as faster wound closure in the mouse model [73]. The researchers proposed that the accelerated wound repair was most likely due to enhanced keratinocyte proliferation in the neo-epidermis and granulation tissue formation. Notably, the effect was potentially mediated by GM-CSF since the concentration of this cytokine was higher in the langerin-depleted mice. Thus, the study by Rajesh and colleagues established that langerin+ cells, i.e., LCs and dermal DCs, probably inhibit keratinocyte proliferation during acute wound healing [73]. Li and colleagues, on the contrary, showed that the loss of CD11c+ cells including LCs and DCs resulted in the failure of wound closure in the murine model [74]. Specifically, in the absence of CD11c+ cells, re-epithelization of acute wounds did not occur, and the wounds remained completely open. The wounds did not heal properly in that case, probably due to reduced cellular proliferation, which was evidenced by the low expression of the Ki67 proliferation marker. Furthermore, the study by Li and colleagues demonstrated that CD11c^+^ cells contributed to the earliest stages of wound repair in part by regulating the expression of IL-22 and IL-23 cytokines since the concentration of these cytokines was low in the CD11c^+^-depleted mice. Overall, LCs and DCs potentially contribute significantly to acute wound healing. However, the exact mechanism and functions of these cells remain to be elucidated.

### 2.2. Adaptive Immunity in Acute Wound Healing

In addition to innate immunity, the cells of the adaptive immune system also participate in acute wound healing. Healthy skin contains an enormous number of T cells—around 20 billion cells—which are represented by innate resident γδ T cells and NKT cells as well as conventional CD4^+^ Th1, Th2, Th17 and CD8^+^ cytotoxic T cells [75,76]. Besides the aforementioned effector cells, there is a population of natural or thymic-derived Foxp3^+^CD25^+^CD4^+^ regulatory T cells, which control the magnitude of immune response and prevent an immune reaction towards self-antigens [77]. Although skin wounds contain ample amounts of T cells, it is generally accepted that T cells are not essential for cutaneous healing to occur [78]. By contrast, T lymphocytes are associated with a pro-inflammatory response, which explains why in the absence of T lymphocytes, the repair of wounds takes place more quickly [78]. However, Wang and colleagues showed the importance of T cells for the regulation of wound resolution [79]. In mice lacking T cells, skin wound closure happened more quickly, but resulted in aberrant healing, namely, excessive inflammation, enhanced scar formation and diminished angiogenesis. On the other hand, the adoptive transfer of CD4^+^ T cells partially restored the normal phenotype by reducing the scar. It is, therefore, still disputable whether or not T cells are crucial for proper wound resolution. The situation is made more complicated by the existence of unconventional γδ T cells, which are very abundant in skin [80]. The lack of skin γδ T cells is associated with late wound resolution and decreased inflammation [81]. Thus, the exact role of T cells in acute wound healing remains complex. Nonetheless, T lymphocytes actively participate in skin injury resolution by interacting with other immune cells and are capable of modulating their response [76,78].

Memory CD4^+^ and CD8^+^ T cells are the first cells to respond to skin injury by the previously exposed antigen, when they undergo proliferation and mediate their secretory and cytotoxic functions [82,83]. In addition to memory cells, resident immune cells start recruiting circulating immune cells, including T cells. The infiltration of T cells to the site of injury is crucial for successful healing of the wound since a delay or impairments in this process result in inadequate healing [59]. Th1 cells are the main T cell subtype that participates in the initial stages of wound healing. Polarization towards Th1 phenotype is mediated by IFN-γ [7,84]. In turn, Th1 cells also express IFN-γ and maintain the pro-inflammatory environment of injured skin by increasing inflammation and reducing tissue repair [78]. In addition to CD4^+^ cells, cytotoxic CD8^+^ T cells are active participants in the initial stages of acute wound healing as well. Early after the onset of a skin injury, DCs migrate to local lymph nodes and present antigens to the naïve CD8^+^ T cells [10]. After antigen recognition, these cells differentiate into effector or cytotoxic T cells and memory CD8+ T cells and relocate to the site of injury. Cytotoxic T cells contribute to the acute wound healing process by releasing microbicidal factors that clear the infection [10]. Besides conventional alpha beta T cells, γδ T cells take part in inflammatory response during wound healing. Specifically, they recognize DAMPs and secrete IL-17 and IL-22, which further promote inflammation [81].

T cells also take part in the later stages of wound healing, including re-epithelization and remodeling. In particular, γδ T cells release IGF-1, which promotes keratinocyte survival and proliferation [80,81]. Furthermore, T cells facilitate polarization of anti-inflammatory macrophages, which in turn suppress inflammation and stimulate ECM deposition, angiogenesis and tissue repair [7]. T cells, specifically Tregs, are also important for the regulation and successful resolution of the immune response. Skin regulatory T cells are represented by the resident cells as well as circulating Tregs, which accumulate in the injured tissue due to the presence of the cutaneous lymphocyte antigen (CLA) and the skin-homing receptor CCR6 [10]. In the skin of adult mice, Tregs comprise 20–60% from the whole CD4^+^ T cells population [85]. Peripheral blood Tregs express proteins, including CLA, CCR6 and CCR4 that facilitate their migration into the skin [86]. Moreover, Tregs suppress inflammation by inhibiting the effector functions of CD4^+^ T lymphocytes, namely, Th1, Th2 and Th17 cells, as well as CD8^+^ T lymphocytes [78]. The importance of Tregs is demonstrated by the study performed by Haertel and colleagues, in which Treg depletion compromised normal acute wound healing. Specifically, it led to impaired re-epithelization, reduced wound closure and delayed vessel maturation [87]. Importantly, there was an increase in the number of αβ TCR expressing T-bet/IFN-γ or RORγt/IL-17A as well as higher levels of IFN-γ, IL-17A and IL-4 in the Treg-depleted wounds. Tregs suppress an immune response via a number of different mechanisms. Those mechanisms include an expression of anti-inflammatory cytokines, such as IL-10, TGF-β and IL-35, as well as secretion of apoptotic factors, granzyme and perforin. Other mechanisms involve the production of CD39/CD73 and regulation of CTLA-4 [88]. In a recent study by Zaiss and colleagues, it was shown that several immune regulatory processes associated with Tregs are mediated via Amphiregulin, an epidermal growth factor (EGF)-like growth factor. For instance, Amphiregulin is responsible for local activation of TGF-β to maintain survival and proliferation of the local Tregs [77].

Besides Tregs, Th2 cells also participate in wound resolution [89]. Specifically, Th2 immunity involves IL-4, IL-5, IL-10 and IL-13 cytokines, which act on other cells and promote wound contraction, angiogenesis and formation of a new epithelial barrier [90]. Another CD4^+^ T lymphocyte type that plays an important role in skin and mucosal wound healing, is Th17. Specifically, Th17 eliminates pathogens and modulates surfaces and adjusts epithelial cell morphology. Certain cytokines activate Th17 cells, including IL-6, TGF-α and IL-1β. Th17 cells, in turn, express TNF-α, IL-17 and IL-22, which enhance survival and proliferation rate and promote skin tissue regeneration [59,91]. Overall, both skin resident and circulating T cells take part in all stages of acute wound healing by inducing inflammation as well as governing its resolution and tissue repair.

In contrast to T cells, much less is known about B cell participation in acute wound healing. In fact, for a long time, the presence of B cells in the skin was doubted and only recently were B cells identified and characterized in the skin of humans and mice [92]. Cutaneous B lymphocytes are located mainly in the dermis and are represented by skin-specific conventional B-2 cells as well as innate B-1 cells. The latter are highly enriched in the skin, compared to blood. The role of B lymphocytes in skin injury repair is also not completely clear. Earlier studies revealed only a modest amount of B cells in acute wounds, concluding that these cells are not important for the process of healing [93]. Later studies, on the other hand, demonstrated that the loss of B cells resulted in a delay of wound repair, while the addition of external B lymphocytes restores normal healing [10]. Altogether, the function of the adaptive immune system in the process of acute wound healing appears to be complicated. Nevertheless, the latest research shows that both T and B cells might be essential for proper resolution of wounds.

## 3. Chronic Wound Healing

The physiological process of wound healing includes four steps: hemostasis, inflammation, proliferation and maturation, whose correct and well-coordinated work ensures proper healing [6]. However, when wounds fail to proceed through this organized process, the healing of skin tissue delays, and this eventually results in chronic wounds. Common features of non-healing wounds are exudation, repeated infection, tissue necrosis, defective re-epithelization, decreased angiogenesis and overproduction of ROS [7,94]. In general, chronic wounds can be categorized into three major categories: diabetic foot ulcers (DFU), vascular ulcers and pressure ulcers [40]. They are usually observed in elderly people suffering from pathological conditions, such as diabetes mellitus, vascular disease and obesity [95]. Diabetes mellitus affects all four steps in skin injury repair [96]. As such, diabetic ulcers are associated with a highly pro-inflammatory profile caused by the excessive expression of inflammatory cytokines, such as TNF-α, and reduced production of pro-healing mediators, including IL-10 and TGF-β. This leads to macrophage polarization towards M1 phenotype, activation and degranulation of CD8^+^ T-cells, resulting in tissue necrosis [97].

**Figure 2:**
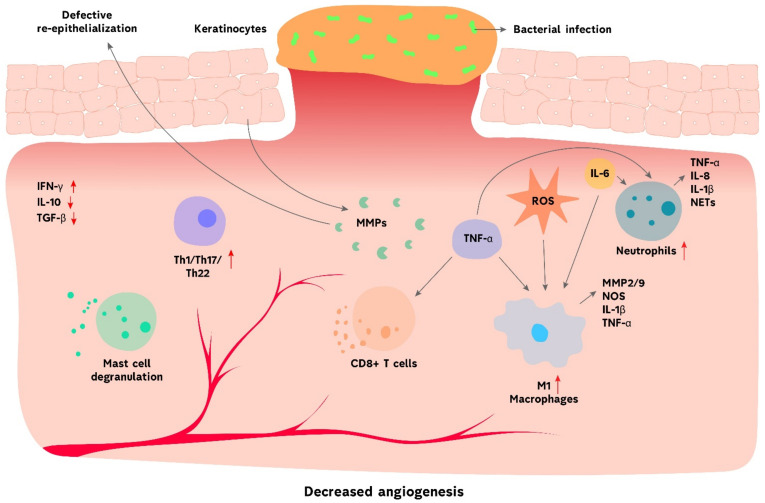
Immune system in chronic wound healing. Common features of chronic wounds are recurrent bacterial infections, decreased angiogenesis, impaired tissue epithelialization and overabundance of ROS. The prolonged presence of neutrophils and M1 macrophages leads to a highly inflammatory profile in the wound. The process is enhanced by mast cells and CD8^+^ T cells’ activity. The level of other inflammatory T cell subtypes, such as Th1, Th17 and Th22, is also increased. Various MMPs, secreted by keratinocytes, contribute to defective re-epithelialization. Together, all those pathological processes promote inflammation, tissue fibrosis and poor vascularization.

Chronic wound healing is characterized by the prolonged presence of myeloid cell populations, such as macrophages, neutrophils and monocytes, in the late stage of inflammation (Figure 2). On the contrary, the percentage of LCs, dermal DCs and eosinophils is reduced throughout the process [98]. Mast cells are also involved in the development of chronic wounds. Cutaneous mast cells are degranulated in diabetic ulcers and the downregulation of their activity accelerates wound repair [99]. T cells take part in maintaining a pro-inflammatory profile of non-healing skin injuries. The ligand for CXCR3, which is found in Th1 cells, is highly expressed in chronic inflammation. Additionally, the level of inflammatory T cell subtypes, such as Th1, Th17 and Th22, is increased in patients with diabetic ulcers [100]. Immune cells actively communicate with non-hematopoietic cells, such as keratinocytes, through the secretion of various signaling molecules [40]. Keratinocytes contribute significantly to the formation of chronic wounds however the precise mechanism is not fully understood. It is known that the impaired regulation of certain miRNAs, such as miR-34a/c, miR-203, miR-19a/b and miR-20a, in keratinocytes impact immune functions and lead to a delayed repair of cutaneous wounds [101,102,103]. Thus, the inhibition of miR-19a/b and miR-20a expression delayed wound healing and induced a stronger inflammatory response in mice via the upregulation of the NF-κB pathway, resulting in overexpression of pro-inflammatory cytokines and chemokines in keratinocytes [101]. The chronic wound formation process is also regulated epigenetically by miRNAs controlling inflammatory responses via the modulation of signaling pathways. Wnt/β-catenin, NF-κB, PI3K/Akt/mTOR, TGF-β/Smad and VEGF pathways are regulated by miRNAs during chronic wound healing [104].

Immune and structural cells actively express and regulate cytokines, chemokines and growth factors during the wound repairing process [100]. For example, an elevated level of IFN-γ, VEGF and soluble VCAM-1, observed in patients with DFU, promotes the healing of ulcers [105]. However, certain factors are dysregulated in non-healing wounds, which is partly responsible for the pathogenesis of the injury. Mice with IL-36 receptor antagonist deficiency showed delayed wound healing due to the overproduction of IL-36γ, TGF-β and CXCL1, redundant infiltration of neutrophils and macrophages and excessive granulation tissue formation [106]. Additionally, the chemokine receptor CCR4 negatively affects chronic wounds caused by diabetes mellitus. CCR4-depleted diabetic mice showed reduced expression of cytokines that promote wound repair, such as IL-6, IL-12, IL-1β, TNF-α and IL-10 [107]. Another study revealed the impaired regulation of transcription factors FOXM1 and STAT3 in patients with DFU. FOXM1 and STAT3 are responsible for the proliferation of macrophages and neutrophils and for their recruitment to diabetic wound microenvironments. Therefore, the inhibition of those factors in DFU patients contributes to the pathogenesis of the disease through the defective recruitment of immune cells [108]. Another factor that facilitates a delay in wound healing is MMP [109]. During normal wound healing, cells in the injured area, such as fibroblasts, keratinocytes and immune cells, are induced by local mediators to secrete MMPs. Those mediators include various cytokines and growth factors involved in wound healing, such as TGF-β, VEGF, EGF, interleukins and interferons [110]. MMPs are normally required in a small amount and are responsible for proper epithelization and proliferation. However, their dysregulation leads to impaired epithelialization and is strongly associated with hard-to-heal wounds (Figure 2) [111]. As such, an increased expression of MMP-9 by activated neutrophils is linked to delayed ulcer repair in diabetic patients [112]. Moreover, high glucose can stimulate the overexpression of MMP-9 via the activation of the ERK/AP1 signaling pathway [113]. Overall, chronic wound healing occurs when the immune system fails to proceed through the normal repairing process, resulting in prolonged presence of neutrophils and pro-inflammatory macrophages in the injured skin, which promotes inflammation, tissue fibrosis and poor vascularization.

### 3.1. Innate Immunity in Chronic Wound Healing

#### 3.1.1. Neutrophils

In regular wound healing, neutrophils are capable of regulating inflammatory response. After completing their functions, neutrophils initiate an apoptotic cell-death pathway, followed by an efferocytosis by macrophages [8]. However, if the process is impaired at any point, it can lead to prolonged presence of neutrophils in the wound environment. Reduced neutrophil apoptosis and an increased level of neutrophil-derived proteases, such as elastase and MMPs, which are known to degrade ECM, as well as neutrophil chemo-attractant CXCL8, are associated with chronic cutaneous wounds [8]. A depletion of neutrophils induced by the injection of 1A8 antibodies into the imiquimod (IMQ)-psoriatic mouse model revealed reduced infiltration of macrophages and CD4^+^ T-cells and decreased production of pro-inflammatory cytokines TNF-α, IFN-γ and IL-1β, indicating the critical role of neutrophils during the wound healing process [114]. Furthermore, MiRNAs play a critical role in delayed wound healing through the regulation of various immune responses. Thus, the deficiency of miR-146a in diabetic mice significantly delays the healing of skin wounds and increases neutrophil infiltration in the injured area via the impaired regulation of IL-1β, TNF-α, IRAK1, TRAF6 and NF-κB pathways [115]. Expression of miR-129 in neutrophils, derived from type 2 diabetic mice and healthy animals, are different. When miR-129-2-3p, which controls Casp6 and CCR2, the genes responsible for inflammatory responses and apoptosis, is overexpressed in neutrophils from type 2 diabetic mice, it facilitates an accelerated repair of diabetic wounds [116].

The level of neutrophil NETs-specific markers, such as citrullinated histone H3 (citH3), is higher in DFU compared to DFU-deficient and healthy patients. It was reported that NETs, released by neutrophils, contributed to delaying wound healing [117]. Thus, the inhibition of NETs may facilitate ameliorated wound healing in diabetic mice. Blocking of peptidyl arginine deiminase 4 (PAD4) by a novel inhibitor, an alginate-GelMa-based hydrogel scaffold, contributed to decreased NETosis and subsequently accelerated wound healing in a diabetic rat model [118]. Moreover, a study reported that neutrophils express the receptor for gonadotropin-releasing hormone (GnRH), the hormone that is associated with worsened wound repair. Treatment of diabetic mice with GnRH agonist promoted a delay in wound healing due to accelerated NETosis caused by the enhanced expression of PAD4 and citH3 [119]. In addition, milk fat globule epidermal growth factor VIII (Mfge8) plays a protective role in diabetic skin wound healing. Mfge8 reduces the NLRP3 inflammasome activation and production of IL-18/IL-1β. Mfge8^−/−^ diabetic mouse model exhibits a higher NETs synthesis, leukocyte infiltration, wound closure failure and impaired vascularization compared to a healthy control [120]. Thus, persistent presence of neutrophils in the wound area delays the healing process through the expression of pro-inflammatory factors, proteases and NETs.

#### 3.1.2. Macrophages

Macrophages are crucial during healing, however their prolonged presence in the injured environment or dysregulation during repair results in impaired wound healing and fibrosis [121]. The failure of macrophages to polarize from a pro-inflammatory M1 towards a pro-healing reparative M2 phenotype is strongly associated with poorly healing wounds (Figure 2) [24]. This failure is due to an overexpression of pro-inflammatory cytokines in the wound microenvironment as well as impaired clearance of apoptotic neutrophils by macrophages [122]. A study comparing macrophages derived from wounds of healthy people to patients suffering from diabetes revealed a distinctive expression of the methyltransferase Setdb2, of which production in wound macrophages is under the control of IFN-β. In diabetic patients, the impaired IFN-β–Setdb2 interaction results in the failure to switch from M1 to M2 phenotype, which leads to the accumulation of pro-inflammatory macrophages in diabetic wounds [123]. Moreover, it was found that M1 macrophages in the diabetic wound microenvironment overexpressed mirRNA-21, which led to the enhanced secretion of inflammatory mediators, such as IL-1α, TNF-α, iNOS, IL-6 and IL-8, and the further polarization of macrophages towards M1 phenotype [124]. However, a strong shift from a pro- to an anti-inflammatory phenotype also undermines the M1-M2 balance. Excessive activity of M2 macrophages during wound healing has been related to the formation of hypertrophic scars [125]. M2 macrophages contribute to scar formation by increasing the synthesis of the ECM proteins as well as secreting MMP-10 and TGF-β1 [126]. In addition, M2 macrophages facilitate fibrotic scarring via the regulation of the Wnt/β-catenin pathway. Late wound macrophages phagocyte SFRP4, the inhibitor of Wnt, and thus promote continuous Wnt activity in injured skin environment, resulting in fibrogenesis rather than regeneration [127,128]. Altogether, a regulation of M1-M2 polarization is crucial for proper wound healing. Any changes in this balance lead to consequences such as non-healed wounds or increased tissue fibrosis.

#### 3.1.3. Innate Lymphoid Cells

Innate lymphoid cells (ILCs) are cells of lymphoid origin and morphology, which do not possess antigen-specific receptors and T and B cell markers but can be activated by innate immune signals [129]. Three lineages of ILCs exert different effector functions depending on its transcription factor and cytokine expression profile [130]. NK cells, which belong to the group 1 ILCs, can produce IFN-γ, granzymes and perforins to eliminate virus-infected cells and cancer cells, distinguishing NK cells from helper ILCs [129]. As a major contributor to IFN-γ production, NK cells are involved in the inflammatory phase of the wound healing process, exerting mostly negative effects on tissue repair [20]. NK cell-derived IFN-γ polarize macrophages to pro-inflammatory M1 phenotype and amplify immune cell infiltration to the wound site by IL-1β, IL-6, IL-12, IL23 and TNF-α expression by macrophages [8]. IFN-γ, mostly secreted by CD4+ T cells, NK and NKT cells, was reported to prevent prolonged neutrophil infiltration in the inflammatory phase and augment fibrosis in the proliferative phase of the wound healing process [131].

ILC2 level is also elevated during chronic cutaneous inflammation. Alarmin cytokines, such as IL-33, TSLP and IL-25, as well as pathogen-associated molecular patterns, can attract ILC2 to the wounded site [129]. In turn, ILC2 were reported to express IL-5, M2 macrophage polarizing IL-13 and IL-4, and amphiregulin—keratinocyte mitogen [40], which also enhances Treg homing to the injury site. Moreover, ILC2s are implicated in stimulation of Treg expansion and Th2 cell polarization [20]. In addition, ILC3 also demonstrates a positive impact on cutaneous wound healing. Upon epidermal Notch1 signal, TNF-α and the ILC3 recruitment chemokines CCL20 and CXCL13 are expressed to recruit ILC3 to the wounded dermis. ILC3 can attract macrophages to the wound bed as a key source of IL-17F and CCL3 and enhance epidermal proliferation by expressing an important keratinocyte growth factor IL-22 [132]. Consequently, ILC3 deficiency was reported to result in diminished macrophage infiltration and epidermal proliferation and delaying cutaneous wound healing in mice [133]. According to previous findings, ILCs roles in wound healing are pleiotropic depending on their subsets. ILC3 and NK cells are implicated in mounting inflammatory responses, while ILC2 can exert an anti-inflammatory effect via enhancing M2 polarization of macrophages and Tregs’ expansion and homing.

### 3.2. Adaptive Immunity in Chronic Wound Healing

The role of adaptive immunity in repairing chronic wounds is not extensively investigated. T cells in chronic wounds are present in a defective and non-responsive state, which is manifested in their inability to secrete factors that they produce in a normal state [7]. T cells take part in maintaining a pro-inflammatory profile of non-healing skin injuries. The ligand for CXCR3, which is found in Th1 cells, is highly expressed in chronic inflammation. Additionally, the level of inflammatory T cell subtypes, such as Th1, Th17 and Th22, is increased in patients with diabetic ulcers [100]. Although Tregs play a balancing role in inflammation by suppressing immune response, some studies showed that an increased number of Tregs in sites of chronic skin inflammation were not only unable to resolve the injury, but even contributed to the pathogenesis of the disease [134,135]. Moreover, skin commensals help to improve chronic wound repair by inducing the activation of T cells with immunoregulatory and tissue-repair functions via a non-classical MHC class I-restricted immune response [136].

Skin-resident B cells are implicated in local antibody production, ectopic lymphoid tissue formation and other effector functions associated with a cutaneous antigen-specific immune response [137]. However, the exact roles of B cell subsets in chronic wound healing still remain to be elucidated. Early conjecture about B cells’ therapeutic impact on cutaneous chronic wounds was provided by Sîrbulescu and colleagues [93], who reported on the acceleration of both acute and chronic wound healing, significant mitigation of apoptosis and improved fibroblast proliferation after topical application of purified mature naïve B cells to the cutaneous wound bed on murine models. As a result, 43% of diabetic wounds were fully closed, compared to 5% in the control group, with no therapeutic effect observed by equivalent amounts of disrupted B cells, hematopoietic stem cells and T cells.

## 4. Modulation of the Immune System to Improve Wound Healing

The hallmark of non-healed wounds is chronic inflammation caused by infiltration of pro-inflammatory immune cells [7]. Therefore, designing strategies based on the regulation of immune cells’ functions is a promising approach in regenerative medicine to treat chronic wounds, including diabetic, vascular and pressure ulcers [138].

### 4.1. M1/M2 Polarization of Macrophages

The modification of macrophage phenotype may become a promising therapeutic approach to treat chronic skin inflammation [125]. Phosphatidylserine-containing liposomes (PSLs) were shown to induce the polarization of macrophages towards M2 phenotype in mice with pressure ulcers (PU). Therefore, treatment with PSLs accelerated wound healing, induced vascularization and inhibited PU formation in an ischemia-reperfusion-induced PU mice model [139]. An overexpression of the scavenger receptor gene CD163, a marker for pro-healing M2 type, in M1 macrophages facilitated a more efficient wound repair, partly through the reduction of MCP-1 expression and production of TGF-α [140]. Exosomes derived from M2 macrophages induce a complete cell reprogramming of classically activated M1 macrophages into a pro-healing phenotype, which promotes cutaneous wound healing. Moreover, M2-derived exosomes secrete paracrine factors, such as IL4, CXCL12 and bFGF, which repair wounds by promoting collagen deposition and re-epithelialization [141]. Furthermore, an expression of long noncoding RNA GAS5 is enhanced in cells from diabetic wounds and contributes to macrophage differentiation toward M1 phenotype via the upregulation of signal transducer and activator of transcription 1. Therefore, GAS5 knockout promotes chronic wound repair by modulating macrophage phenotypes in chronic cutaneous wounds [142].

The inhibition of mineralocorticoid receptor (MR) expression promotes acceleration of the wound healing process in diabetic mice, with no impact on healthy mice wounds. This effect is caused by a reduced production of LCN2, the ligand for MR, which may contribute to macrophage polarization, neo-vascularization and prevention of inflammation [143]. NK-4 is another promising agent for anti-chronic wound therapy. NK-4 is a cyanine dye, which was shown to activate differentiation of tumor-derived macrophages into M1 pro-inflammatory phenotype and stimulate their phagocytic activity. Interestingly, NK-4 treated M1 macrophages, when incubated together with apoptotic Jurkat E6.1 (Apo-J) cells, switched into M2 phenotype through the downregulation of TNF-α secretion and stimulation of IL-10 production [122]. Finally, treatment of diabetic mice with docosahexaenoic acid significantly improved the healing of ulcers by stimulating macrophage polarization toward M2 phenotype [144]. Overall, the polarization of macrophages toward an anti-inflammatory type significantly improves delayed wound healing. Therefore, various approaches for modifying the macrophage phenotype can be used as therapies to treat chronic wounds.

### 4.2. MiRNAs

MiRNAs are important regulators of the immune system. In particular, they control the response of macrophages, monocytes, DCs, granulocytes, mast cells, T cells and other immune cells by regulating the expression of multiple genes [145]. On the other hand, certain miRNAs, such as miR-21, miR-424, miR-31, miR-221 and miR-222, are dysregulated in chronic wounds, leading to improper immune response compared to normal wounds [146]. Thus, post-transcriptional modulation of gene expression of immune cells by restoration of the normal miRNA expression by up- and/or down-regulation of miRNAs can be considered as a rational complementary immunomodulatory therapy to improve chronic wound healing. This may include the delivery of miRNAs embedded into viral vectors, liposomes or biomaterials directly to the wound. As such, miR-146a is a potential target to promote faster wound healing [147]. The knockout of miR-146a significantly delayed skin wound healing in diabetic mice in comparison to diabetic mice without removal of the miR-146a gene by increasing the inflammatory response [115]. Thus, treatment with (2E,6E)-2,6-bis(2-(trifluoromethyl)benzylidene)cyclohexanone (C66) enhanced the secretion of miR-146a and suppressed NF-κB activity in skin injuries of diabetic mice, resulting in an anti-inflammatory response [148]. Moreover, recently, miR-146a was conjugated to cerium oxide nanoparticles (CNP) and applied directly to the diabetic wound. CNP-miR146a accelerated chronic wound healing by decreasing inflammation and increasing angiogenesis [149].

MiRNA-21 is well-known to participate in the chronic healing process [150]. The level of miRNA-21-3p is decreased in diabetic patients compared to healthy ones and treatment with the miR-21-3p agonist stimulated fibroblast activation through the reduction of SPRY1 [151]. Therefore, the overproduction of miRNA-21-3p is another target for DFU treatment. In general, modification of miRNAs expression is a novel target for the treatment of chronic wounds.

### 4.3. Cytokines/Growth Factors and Inhibitors

In addition to regulating the inflammatory phase in wound healing and its resolution, novel approaches relying on transplanting stem cells and allogeneic skin grafts and substitutes highlight the importance of immunomodulation for cutaneous wound healing. Several immunomodulatory approaches targeting both inflammatory factors, such as TNF-α, IL- 1, NF-κB pathway, as well as anti-inflammatory factors, such as IL-4, IL-10 and TGF-β, were applied for tissue repair and regeneration [20,152]. TNF-α, which is implicated in neutrophil and macrophage recruitment, plays an early regulatory role in the inflammatory phase of wound repair. Ritsu and colleagues reported an early expression of TNF-α in full-thickness skin wounds in mice [153]. Moreover, the authors demonstrated delayed wound closure, decreased inflammatory cell and fibroblast number in the wound bed upon anti-TNF-α mAb treatment, as well as accelerated wound healing by delivery of TNF-α. In addition, hyperglycemia can induce TNF-α production by M1 macrophages, and TNF-α levels are elevated in cutaneous wounds in diabetic rats. Applying neutralizing TNF-α antibody and TNF-α antagonist increased keratinocytes’ migratory capacity and accelerated skin wound healing in diabetic rats, respectively [154].

IL-1 family is another important therapeutic target for chronic wound healing. IL-1β is an upstream regulator of inflammasome activity in wound macrophages, which prevents their polarization towards an anti-inflammatory phenotype. Utilizing IL-1 receptor antagonist (IL-1Ra) can exert an immunomodulatory effect by halting IL-1 signaling. Anakinra, a recombinant IL-1R antagonist, was shown to accelerate diabetic wound healing by lowering macrophage and neutrophil infiltration [155]. In addition, Tan and colleagues reported a diabetic wound closure delay by the IL-1–IL-1R1 axis, which could be overcome by engineered ECM-binding IL-1Ra [156]. An inhibition of molecules that are overproduced in chronic wounds, and thus further complicate the healing process, is another approach. Thus, the selective inhibition of MMP-9, that is over-secreted in diabetic ulcers by recruited neutrophils, with (R)-ND-336 inhibitor, accelerated delayed wound healing, making MMP-9 a potential target for the therapy [112].

Delivery of exogenous growth factors can be applied as a treatment of chronic wounds due to their functions in enhancing granulation tissue formation, stimulating angiogenesis and immunomodulation. Growth factors, levels of which were found to be lowered in non-healing wounds, such as PDGF, FGF and EGF, were prime candidates for delivery. For example, PDGF is a crucial factor in wound healing, which is involved in chemoattraction of neutrophils, monocytes and fibroblasts to the injury site in the early stages of wound healing and implicated in the contraction of collagen matrices in the proliferation stage [157]. Accordingly, Regranex^®^—recombinant human PDGF-BB embedded in the gel—was approved by the FDA and EMA for topical application in DFU. Although Regranex^®^ had demonstrated efficacy in treating ulcers in placebo-controlled trials, the elevated risk of adverse events detected during the post-marketing surveillance period led to the withdrawal of Regranex^®^ from the European market [158]. In addition, the FGF family consists of several growth factors implicated in wound healing, such as FGF-2, keratinocyte growth factor-2 (KGF-2), FGF-7 and others. For instance, FGF-2 (also referred to as bFGF) delivered in hydrogel, cryogel, coacervate and biofilm formulations can accelerate wound healing by increasing fibroblast proliferation, organizing collagen deposition, promoting angiogenesis and keratinocytes migration and reducing pro-inflammatory factors such as TNF-α and IL-6 [21]. Therefore, recombinant forms of bovine and human bFGF, such as Fiblast Spray^®^, were used for treatment of burn, pressure and DFU. In another study, polysaccharide hemostasis microspheres were used to mediate the sustained release of KGF-2 in a full-layer skin cutting model in rats [159]. The treatment reduced inflammation and accelerated wound healing, which was attributed to greater bioavailability and controlled release. Altogether, the regulation of immune cells, by modulating the expression of various cytokines and the delivery of growth factors, is a promising strategy in anti-chronic wound therapy.

### 4.4. Stem Cells

Therapies based on stem cell technologies are now being actively investigated in the field of regenerative medicine [160]. Experimental preclinical and clinical studies showed the positive impact of adult stem cells’ treatment on chronic wound healing [161]. This beneficial effect is due to the unique properties of stem cells to suppress immune response, repair injured tissue and support the local cells via the secretion of trophic and paracrine factors [162]. Various in vivo studies demonstrated the well-organized interaction between stem cell secretomes and mediators found in the wound area, which resulted in a lesser scar size and reduced inflammation as well as improved re-epithelialization of damaged tissue [163].

Stem cell-secreted exosomes possess similar biological activity as stem cells and thus are also a potential target to treat chronic wounds. The immunomodulatory properties of exosomes can possibly be activated by an inflammatory environment. Adipose stem cell-derived exosomes when treated with pro-inflammatory cytokines, such as IFN-γ and TNF-α, enhance their immunosuppressive properties and switch the polarization of macrophages towards M2 phenotype [164]. The treatment of wounds with adipose stem cell-derived microvesicles significantly increased the production of VEGF, PDGFA, EGF and FGF2, and thus enhanced epithelialization, angiogenesis and collagen deposition, eventually leading to accelerated wound healing [161]. Adipose-derived stem cell-secreted exosomes reduced ulcer size in diabetic mice by promoting angiogenesis, granular tissue formation, immunosuppression and reduction of oxidative stress-related proteins [165]. In addition, exosomes from human urine-derived stem cells were shown to overexpress the pro-angiogenic factor DMBT1 and thus promote angiogenesis in diabetic mice [166]. Lipoma-derived stem cells are another target in regenerative medicine. An in vitro study revealed that lipoma-derived stem cell secretomes activate other cells, such as macrophages, and stimulate overexpression of IL-10, while suppressing the production of TNF [167]. Taken together, due to their immunosuppressive functions, stem cells and their secretomes can serve as potential tools for regenerative medicine to treat chronic wounds.

### 4.5. Negative Pressure Wound Therapy

In addition to delivery of biologics, physical treatment approaches such as hyperbaric oxygen therapy, low-level laser therapy and electrical stimulation were attempted to accelerate the healing of chronic ulcers, although with inconsistent success [168]. Negative pressure wound therapy (NPWT), also known as vacuum-assisted closure, is another physical treatment approach, which is based on differential suction or vacuum creation onto the wounds to enhance fluid removal, reduce edema and alter the wound microenvironment [169]. NPWT demonstrated promising results in accelerating the healing of lower-limb ulcers of vascular origin, such as DFU. Meta-analysis of randomized controlled trials performed by Lui and colleagues assessed safety, cost effectiveness and therapeutic efficacy of NPWT in the treatment of DFU [170]. Analysis of 11 trials led to the conclusion that NPWT has a higher rate of complete ulcer healing, more rapid healing of wounds and significant reduction of wound surface area compared to standard dressings, with no significant effect on adverse events. In another systematized review, Wynn and Freeman also reported better clinical outcomes upon NPWT compared to standard treatment of DFU [171]. Although both reviews included limitations such as methodological flaws in studies and inconsistent NPWT protocols, current evidence supports the efficacy of the vacuum-assisted closure approach as a non-invasive adjunctive treatment of chronic wounds. Precise underlying molecular mechanisms of enhancing wound healing by NPWT are not fully understood. However, alterations in cytokine expression and attraction of immune cells refer to immunomodulatory effects exerted by NPWT. For example, NPWT was shown to halt amplification of wound inflammatory stimuli by preventing iNOS-mediated excessive NO production. In addition, NPWT prevents NF-κB activation by IkB-α inhibition and ATF-3 upregulation, subsequently reducing the expression of pro-inflammatory cytokines such as TNF-α and IL-6 [172]. NPWT was reported to reduce iNOS, IL-6 and TNF-α by regulating the MAPK-JNK pathway [173]. NPWT enhanced healing of full-thickness skin defects in diabetic mice by lowering the number of CD68+ macrophages, reducing the level of pro-inflammatory cytokines TNF-α, IL-6, IL-1β and suppressing autophagy [174]. These observations emphasize the role of immunomodulatory effects in the therapeutic mechanism of accelerating chronic wound healing by negative pressure wound therapy. Table 1 summarizes the various approaches for immunomodulation to improve wound healing.

## 5. Conclusions

A number of studies showed the critical role of immune cells in all four steps of tissue repair, including re-epithelialization and tissue remodeling. It has also been shown that the impaired regulation of their functions contributes to the formation of chronic wounds and fibrotic scar. An attempt to modulate the immune system in order to improve wound healing has been successfully tested by many research groups. In this review, we discussed some of these immunomodulatory strategies, namely, macrophage polarization, regulation of miRNA expression, inhibition of pro-inflammatory cytokines and treatment with anti-inflammatory cytokines. These strategies are frequently used in combination with other methods to improve their efficiency. In particular, bioactive agents can be incorporated into biomaterials such as nanoparticles and cryogels, which promote their protection and controlled release. Despite positive results obtained in pre-clinical studies, there are multiple issues that must be considered before the translation of the aforementioned treatments to clinical trials. First and foremost, animal models in which the treatments have been tested are unable to fully reproduce the complexity of human chronic wounds. Thus, the immunity of wounds, as well as the process of healing in humans, are different to wound pathogenesis in mice, which are most commonly used for skin injury research. Secondly, strategies that modulate the immune system must be very carefully assessed in terms of safety because they could induce an improper immune response. For instance, these treatments can impact the immune response in other locations besides wounds and affect immune conditions present in patients, such as infections, allergies and autoimmune diseases. Finally, technical considerations of the potential treatments should be considered, such as delivery methods and application techniques as well as timing of the therapy. Overall, although immunomodulatory therapies showed promising results in pre-clinical studies, their transition to clinical trials may be challenging due to the complexity of chronic wounds in humans and therefore, additional studies should be performed.

## Figures and Tables

**Table 1:** Modulation of the immune system to improve wound healing.

Immunomodulation Strategy	Treatment	Wound Type	Mediated Effects	Reference
M2 macrophage polarization	Phosphatidylserine-containing liposomes	Pressure ulcer in young and middle-aged mice	Prevented pressure ulcer formation, promoted wound healing and enhanced ECM deposition and angiogenesis	[139]
	Exosomes derived from M2 macrophages	Acute skin wounds in a murine model	Accelerated primary as well as complete wound closure; enhanced re-epithelization, ECM formation and angiogenesis	[141]
	Knockdown of long non-coding RNA *GAS5*	Diabetic wounds in a murine model	Accelerated wound closure	[142]
	Topical pharmacological blockade of the mineralocorticoid receptor	Diabetic wounds in a murine model	Accelerated wound closure and improved angiogenesis; suppressed inflammation	[143]
	Docosahexaenoic acid	Diabetic wounds in a murine model	Faster wound healing; increased vessel density and perfusion; alleviated inflammation	[144]
microRNA overexpression/stimulation	microRNA-146a overexpression with a synthetic curcuminoid analog	Diabetic wounds in a murine model	Enhanced wound closure, faster re-epithelialization, suppressed inflammatory mediators	[148]
	Cerium Oxide Nanoparticles Conjugated with MicroRNA-146a	Diabetic wounds in murine and porcine models	Accelerated wound closure, increased strength and elasticity in a murine model; improved wound healing, increased angiogenesis and reduced inflammation in a porcine model	[149]
	Human keratinocyte-derived microvesicles expressing microRNA-21	Diabetic wounds in a rat model	Rapid wound closure, increased angiogenesis and enhanced fibroblast differentiation	[150]
	miR-21-3p agonist	Diabetic wounds in a murine model	Accelerated wound healing and enhanced fibroblast activity	[151]
Pro-inflammatory cytokine inhibition	Etanercept, a TNF-α neutralizing peptide	Diabetic wounds in a rat model	Improved wound healing and closure	[154]
	IL-1R antagonist	Diabetic wounds in a murine model	Reduced inflammation, decreased neutrophil and macrophage infiltration, accelerated wound closure	[155]
Growth factors	PDGF-BB	Ulcers in diabetic patients	Attracted neutrophils and macrophages into the wound; stimulated fibroblast recruitment and proliferation; enhanced collagen synthesis and ECM deposition; and accelerated wound healing	[158]
	KGF-2	A full-layer skin cutting model in rats	Suppressed inflammation and accelerated wound healing	[159]
Stem cells and microvesicles	Human adipose stem cell-derived microvesicles	Full-thickness cutaneous wound models in mice	Accelerated wound closure, enhanced re-epithelialization, collagen deposition and angiogenesis, increased cell proliferation	[161]
	Human adipose stem cell-derived microvesicles	Diabetic skin wound models in rats	Enhanced formation of granulation tissue, increased angiogenesis, greater expression of growth factors, decreased inflammatory and oxidative factors	[165]
	Exosomes from human urine-derived stem cells	Full-thickness excisional skin wounds in diabetic mice	Accelerated wound healing and enhanced angiogenesis	[166]
	Lipoma-derived stem cells	In vitro “scratch” wound assay	Increased fibroblast migration and wound closure	[167]
Negative pressure wound therapy	Vacuum-assisted closure (VAC) therapy system	Human diabetic foot ulcers	Attenuated inflammation, increased ECM formation, decreased the expression of TNF-α, IL-6 and iNOS	[172]
	Vacuum-assisted closure (VAC) therapy system	Human diabetic foot ulcers	Suppressed wound inflammation, decreased levels of IL-6, iNOS, TNF-α and P-c-Jun N-terminal kinase	[173]
